# Supplementary material for: Interface-based tuning of Rashba spin-orbit interaction in asymmetric oxide heterostructures with 3d electrons
Source: Nat Commun. 2019 Jul 11;10:3052. doi: 10.1038/s41467-019-10961-z (PMC6624272; doi:10.1038/s41467-019-10961-z)
Supplement: Supplementary file 1 — Supplementary Information [file 41467_2019_10961_MOESM1_ESM.pdf]

Supplementary information

Interface-based tuning the Rashba spin-orbit interaction in asymmetric oxide heterostructures with 3d electrons

Lin *et al.*

Supplementary Note 1: Spin splitting of $SrTiO_3$//$LaAlO_3$ (STO//LAO) heterostructure

The schematic structure of conventional $SrTiO_3$//$LaAlO_3$ (STO//LAO) is shown in the Supplementary Fig. 1, which is conducting at the interface. Due to the confinement effect of the interface, the degeneracy of the 3*d* orbitals is lifted with the d_{xy} orbital located below $d_{yz/xz}$ orbitals (Supplementary Fig. 1b). Considering the spin orbit interaction, the degeneracy of the $d_{yz/xz}$ orbitals are further lifted. As a result of the naturally broken inversion symmetry at the interface, the double degeneracy of all orbitals are lifted, i.e. spin splitting. Similar to the LAO//STO/LAO structure, the spin splitting energy can also be tuned by filling carriers, as shown in Supplementary Fig. 1c.

However, there are differences of spin splitting in STO//LAO and LAO//STO/LAO structures. First, the lowest 3*d* orbital of LAO//STO/LAO is $d_{xz/yz}$ orbital due to the biaxial strain effect, whereas it is d_{xy} orbital for STO//LAO heterostructure ascribed to the confinement effect, shown in Supplementary Fig. 2. Second, the transition from the cubic Rashba term to linear occurs around 0.01 e/Ti in trilayers structure while it is around 0.13 e/Ti for STO//LAO bilayer structure.

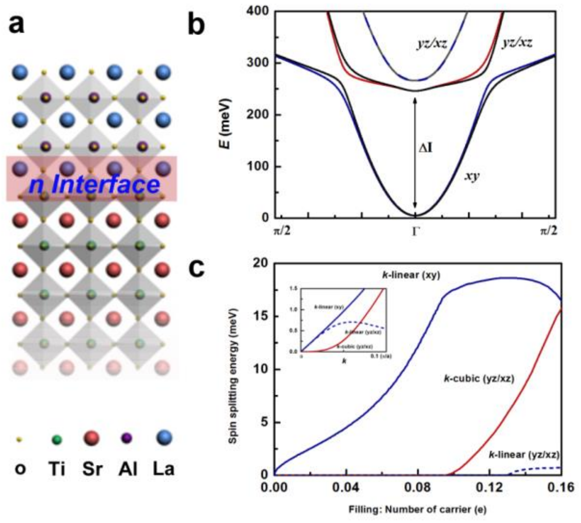

Supplementary Figure 1 | Spin splitting of $SrTiO_3$//$LaAlO_3$ (STO//LAO) heterostructure. (a) Schematic structure of the STO//LAO heterostructure. (b) Band structure of t_{2g} orbitals in the

STO//LAO asymmetric heterostructure. The interface effect lifts the t_{2g} degeneracy, resulting in the splitting (**Δ_I**) between d_{xy} and $d_{yz/xz}$ (d_{xy} is the lowest band, while $d_{yz/xz}$ is lowest in the LAO//STO/LAO heterostructure). The degeneracy of the $d_{yz/xz}$ orbital is further lifted by spin orbit interaction. (c) Spin splitting energy of the t_{2g} orbitals as function of carriers filling in the STO/LAO heterostructure. The inset shows the **k**-linear and cubic spin splitting of the corresponding orbitals.

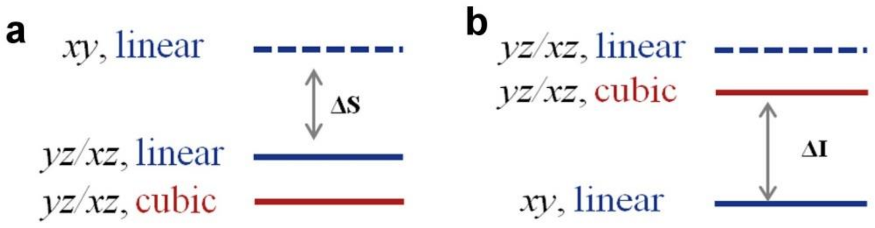

Supplementary Figure 2 | Lifting of the t_{2g} orbital degeneracy. (a) and (b) are for LAO//STO/LAO and STO//LAO heterostructures, respectively.

Supplementary Note 2: Growth and quality of the LAO//STO/LAO heterostructure

To achieve the AlO_2 terminated surface, the LAO substrates were annealed prior to PLD at 1050 °C in the air for 10 h[1]. The single termination can be expected first from the AFM topography with obvious and homogeneous steps, and further confirmed by the terrace structure with step around 4 Angstrom (Å) (Supplementary Fig. 3). Before the deposition of STO, a LAO buffer layer of 10 unit cell (uc) was deposited to improve the surface smoothness. A STO layer with controlled thickness was then deposited, which was followed by the deposition of a 10 uc LAO top layer. Supplementary Fig. 4a shows a typical RHEED pattern for the LAO//STO/LAO (10//15/10 uc). Then the gown films were characterized by X-ray diffraction technique: X-ray reflectivity data was used to double confirm the thickness of the grown films (Supplementary Fig.4b), whereas the theta-2theta measurements were utilized to extract the lattice constant of ***c*** of

STO (Supplementary Fig.4c and d). Furthermore, the quality of the grown film was characterized by STEM technique, as shown in Supplementary Fig. 5. The HAADF and strain maps reveals fully strained nature of the grown films.

Here we would like to discuss about the anomalous trend of the ***c*** lattice constant of STO in the LAO//STO/LAO structure. As shown in Supplementary Fig. 4d, the *c* decreases from 3.946 to 3.895 Å upon the decreasing of the STO thickness in the series of heterostructures. For such epitaxial films, the compressive strain from the LAO substrate due to the lattice mismatch should remain constant, and the *c* value for a 40 uc STO film prepared under similar conditions, but without the LAO capping layer, was reported to be 3.96 Å [1]. This lattice constant is also confirmed by our first-principle calculation, indicated in Supplementary Fig. 4d by the upper dash line. Therefore, the strain from the LAO substrate alone cannot explain the observed variation of the STO lattice parameter. This means that, besides the elastic deformation due to the LAO substrate, there exists an extra stress that compresses the STO layer in the out-of-plane direction. If this thickness-dependent variation of *c* was caused by relaxation of the STO layer, the opposite trend would be expected; so with the interdiffusion effect, which would elongate the out-of-plane lattice constant of STO near the interface[2]. More importantly, the value of *c* closely follows the quadratic relationship with the STO thickness, as shown in Supplementary Fig.4d. With the assumptions that *c* is proportional to the extra strain, and the related electric field is in a linear relationship with the STO thickness, the good fit suggests the existence of an electrostrictive effect in the STO layers, analogous to the observation made in LAO/STO heterostructures[3]. The decrement of *c* lattice constant is also confirmed by our first-principle calculation, indicated in Supplementary Fig. 4d.

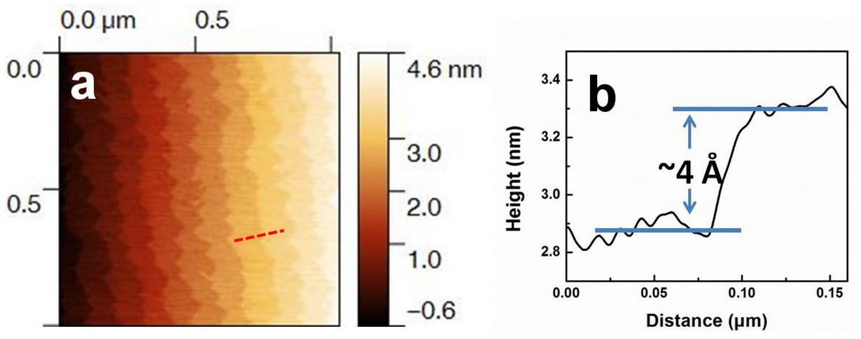

Supplementary Figure 3 | Treated $LaAlO_3$ (LAO) substrate. (a) The AFM topography for the LAO substrate after treatment. (b) shows the line profile indicated by the red dash line in (a).

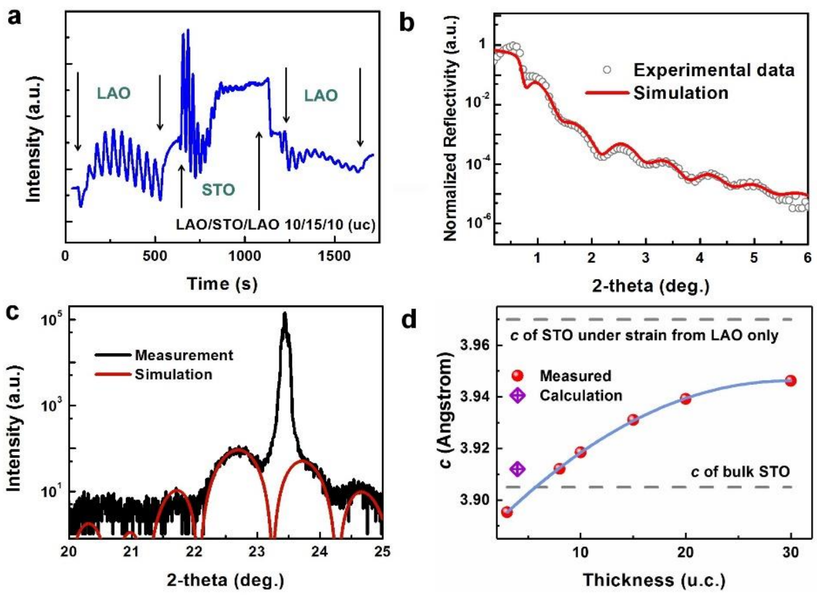

Supplementary Figure 4 | Growth of the $LaAlO_3$//$SrTiO_3$/$LaAlO_3$ (LAO//STO/LAO) heterostructue. (a) RHEED oscillation observed during the film deposition of the LAO//STO/LAO (10//15/10 uc) heterostructure. (b) X-ray reflectivity data for the same sample. The symbols correspond to the experimental data and the curve the simulation result. (c) Theta-2theta data measured for the LAO//STO/LAO heterostructure with 10 uc STO, where the red curve is obtained through fitting. (d) The measured out-of-plane lattice constant *c* (red solid circle) as function of the STO layer thickness, while the purple square shows the *c* lattice constant from first-principle calculation. The dash curves indicates the *c* values of bulk STO and STO thin film under the strain from LAO, and the solid line is to guide the eyes.

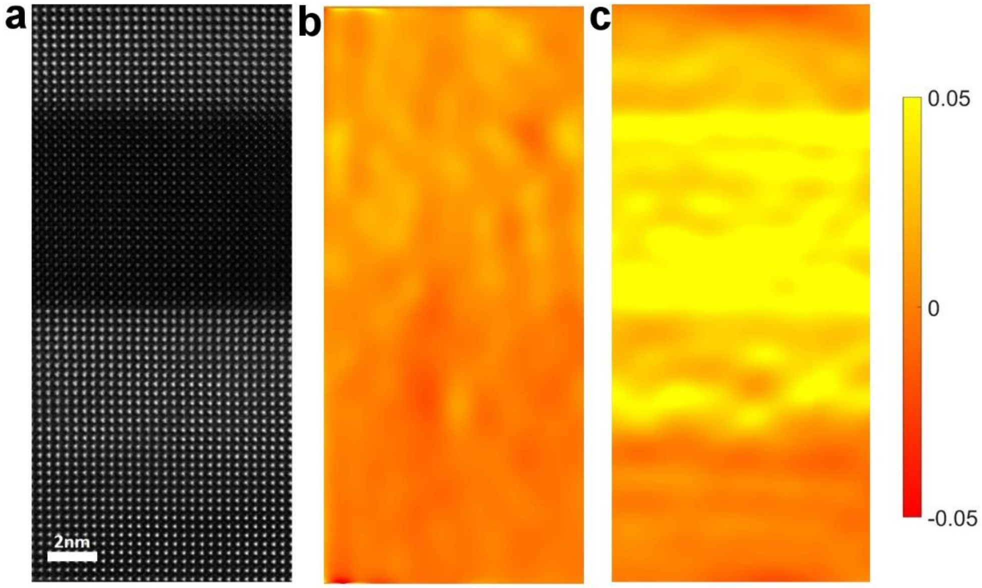

Supplementary Figure 5 | Strain state of the $LaAlO_3$//$SrTiO_3$/$LaAlO_3$ (LAO//STO/LAO) heterostructure. (a) HAADF images of LAO//STO/LAO heterostructure. (b) and (c) show the strain analysis maps of the strain components parallel (ε_{xx}) and perpendicular (ε_{yy}) to the interface, respectively. The homogeneous distribution of ε_{xx} indicates the identical in-plane lattice constant was maintained during the film grown, while around 4% of ε_{yy} in the STO layer is consistent with the XRD data in Supplementary Fig. 4d.

Supplementary Note 3: Density functional theory calculations of the out-of-plane lattice constant *c*

Density functional theory calculations of out-of-plane lattice constant ***c*** are carried out with the Vienna ab initio simulation package (VASP)[4,5] within the projector augmented-wave method. A kinetic energy cutoff of 500 eV is used and the Brillouin zone of $SrTiO_3$ is sampled with 16×16×16 *k*-point grid. We use the revised Perdew-Burke-Ernzerhof generalized gradient approximation for solid (PBEsol) of the exchange-correlation potential[6]. The calculated *c* of bulk $SrTiO_3$ is 3.910Å, in a good agreement with the experimental value of 3.905 Å. To simulate the compressive strain effect from $LaAlO_3$ substrate, we fix the in plane lattice constant of STO at

a_{LAO}=3.80 Å and then relax the out-of-plane lattice constant as well as internal atomic positions. The optimized ***c*** of $SrTiO_3$ is 3.970Å, as shown in Supplementary Fig. 4d, which is comparable to experimental value 3.96Å[1].

We further model our $LaAlO_3$//$SrTiO_3$/$LaAlO_3$ by a $(LaAlO_3)_n/(SrTiO_3)_n$ superlattice containing n=4 layers each of $LaAlO_3$ and $SrTiO_3$ with alternating LaO/TiO_2 and AlO_2/SrO interfaces. As shown in our previous study[7], the polar instability in the heterostructure will induce a large build-in electric field, leading to formation of oxygen vacancies at the AlO_2/SrO interface. We construct a 2×2 lateral supercell and remove one oxygen atom to simulate the oxygen vacancy. The Brillouin zone of $SrTiO_3$ is sampled with 8×8×2 k-point grid. The in plane lattice constant is fixed at $2\times a_{LaAlO3}$=7.60Å, and the out-of-plane lattice constant as well as internal atomic positions are relaxed. We then average the distance of Sr atoms along c direction and obtain the effective out of plane lattice of $SrTiO_3$ is c=3.91Å, in an agreement with experimental measurement. This value is much smaller than the c=3.970Å of $SrTiO_3$ under compressive strain from LAO substrate, in consistent with the electrostriction scenario.

Supplementary Note 4: Fitting the magnetoconductance by ILP theory

The ILP theory is a comprehensive description of weak localization/antilocalization due to spin orbit interaction, and has been often used in literature however, we have not seen the equation for full interaction presented explicitly. Here, we derive the equation used in this research. In calculation of linear and cubic components of Rashba, we have utilized ILP theory and notation as outlined in Ref 8. The conductance due to spin-orbit interaction (SOI) is given by:

$$\Delta\sigma(B) = -\frac{e^2}{4\pi^2\hbar}\left[\frac{1}{a_0} + \frac{2a_0+1+\frac{B_{so1}}{B}+\frac{B_{so3}}{B}}{a_1\left(a_0+1+\frac{B_{so1}}{B}+\frac{B_{so3}}{B}\right)-2\frac{B_1}{B}} + \Psi\left(0.5+\frac{B_\phi}{B}\right) + 2\ln\left(\frac{B_{tr}}{B}\right) - \sum_{n=1}^{\infty}\left(\frac{3}{n} - \frac{3a_n^2+2a_n\left(\frac{B_{so1}}{B}+\frac{B_{so3}}{B}\right)-1-2(2n+1)\frac{B_{so1}}{B}}{\left(a_n+\frac{B_{so1}}{B}+\frac{B_{so3}}{B}\right)a_{n-1}a_{n+1}-2\frac{B_{so1}}{B}[(2n+1)a_n-1]}\right)\right], \qquad (1)$$

where Ψ is the digamma function, B_{so1}, B_φ and B_{so3} refer to characteristic fields of linear SOI, phase coherence and cubic SOI, respectively and

$$\mathrm{a_n} = \mathrm{n} + \frac{3}{2} + \frac{B_{so1}}{B} + \frac{B_{so2}}{B} + \frac{B_{so3}}{B}.$$

Since this equation includes both field dependent and field independent components to the conductance, zero field contribution,

$$\Delta\sigma(0) = \frac{e^2}{4\pi^2\hbar}\left[-0.5\ln\left(\frac{\tau_1}{\tau_2}\right) + \ln\left(\frac{\tau_1}{\tau_2}+\frac{\tau_1}{\tau_{S_x}}\right) + 0.5\ln\left(\frac{\tau_1}{\tau_2}+\frac{\tau_1}{\tau_{S_z}}\right)\right]$$

$$= \frac{e^2}{4\pi^2\hbar}\left[-0.5\ln\left(\frac{B_2}{B_{tr}}\right) + \ln\left(\frac{B_2}{B_{tr}}+\frac{B_x}{B_{tr}}\right) + 0.5\ln\left(\frac{B_2}{B_{tr}}+\frac{B_z}{B_{tr}}\right)\right], \qquad (2)$$

needs to be subtracted. The zero field component can be re-written by introducing a parameter α for argument of the logarithm unitless.

$$\Delta\sigma(0) = \frac{e^2}{2\pi^2\hbar}\left[-0.5\ln\left(\frac{\alpha B_2}{\alpha B_{tr}}\right) + \ln\left(\frac{\alpha B_2}{\alpha B_{tr}}+\frac{\alpha B_x}{\alpha B_{tr}}\right) + 0.5\ln\left(\frac{\alpha B_2}{\alpha B_{tr}}+\frac{\alpha B_z}{\alpha B_{tr}}\right)\right]$$

$$= \frac{e^2}{2\pi^2\hbar}\left[-0.5\left\{\ln\left(\frac{B_2}{\alpha}\right) - \ln\left(\frac{B_{tr}}{\alpha}\right)\right\} + \left\{\ln\left(\frac{B_2}{\alpha}+\frac{B_x}{\alpha}\right) - \ln\left(\frac{B_{tr}}{\alpha}\right)\right\} + 0.5\left\{\ln\left(\frac{B_2}{\alpha}+\frac{B_z}{\alpha}\right) - \ln\left(\frac{B_{tr}}{\alpha}\right)\right\}\right]$$

$$= \frac{e^2}{2\pi^2\hbar}\left[-0.5\ln\left(\frac{B_2}{\alpha}\right) + \ln\left(\frac{B_2}{\alpha}+\frac{B_x}{\alpha}\right) - \ln\left(\frac{B_{tr}}{\alpha}\right) + 0.5\ln\left(\frac{B_2}{\alpha}+\frac{B_z}{\alpha}\right)\right]$$

$$= \frac{e^2}{4\pi^2\hbar}\left[-\ln\left(\frac{B_\phi}{\alpha}\right) + 2\ln\left(\frac{B_\phi}{\alpha}+\frac{B_{so1}+B_{so3}}{\alpha}\right) - 2\ln\left(\frac{B_{tr}}{\alpha}\right) + \ln\left(\frac{B_\phi}{\alpha}+\frac{2B_{so1}+2B_{so3}}{\alpha}\right)\right], \qquad (3)$$

The contribution only from field dependent component of the weak antilocalization is:

$$\Delta\sigma(B)-\Delta\sigma(0)=-\frac{e^2}{4\pi^2\hbar}\left[\frac{1}{\mathrm{a}_0}+\frac{2\mathrm{a}_0+1+\frac{\mathrm{B_{so1}}}{\mathrm{B}}+\frac{\mathrm{B_{so3}}}{\mathrm{B}}}{\mathrm{a}_1\left(\mathrm{a}_0+1+\frac{\mathrm{B_{so1}}}{\mathrm{B}}+\frac{\mathrm{B_{so3}}}{\mathrm{B}}\right)-2\frac{\mathrm{B_{so1}}}{\mathrm{B}}}+\Psi\left(0.5+\frac{\mathrm{B_\phi}}{\mathrm{B}}\right)+2\ln\left(\frac{\mathrm{B_{tr}}}{\mathrm{B}}\right)-\ln\left(\frac{\mathrm{B_\phi}}{\alpha}\right)+\right.$$

$$2\ln\left(\frac{\mathrm{B_\phi}}{\alpha}+\frac{\mathrm{B_{so1}}+\mathrm{B_{so3}}}{\alpha}\right)-2\ln\left(\frac{\mathrm{B_{tr}}}{\alpha}\right)+\ln\left(\frac{\mathrm{B_\phi}}{\alpha}+\frac{2\mathrm{B_{so1}}+2\mathrm{B_{so3}}}{\alpha}\right)-\sum_{\mathrm{n}=1}^{\infty}\left(\frac{3}{\mathrm{n}}-\right.$$

$$\left.\left.\frac{3\mathrm{a_n^2}+2\mathrm{a_n}\left(\frac{\mathrm{B_{so1}}}{\mathrm{B}}+\frac{\mathrm{B_{so3}}}{\mathrm{B}}\right)-1-2(2\mathrm{n}+1)\frac{\mathrm{B_{so1}}}{\mathrm{B}}}{\left(\mathrm{a_n}+\frac{\mathrm{B_{so1}}}{\mathrm{B}}+\frac{\mathrm{B_{so3}}}{\mathrm{B}}\right)\mathrm{a_{n-1}}\mathrm{a_{n+1}}-2\frac{\mathrm{B_{so1}}}{\mathrm{B}}[(2\mathrm{n}+1)\mathrm{a_n}-1]}\right)\right], \qquad (4)$$

Since α is a free parameter, to cancel terms related to B_{tr}, we set it to field, $\alpha=B$, then the full equation becomes:

$$\Delta\sigma(B)-\Delta\sigma(0)=-\frac{e^2}{4\pi^2\hbar}\left[\frac{1}{a_0}+\frac{2a_0+1+\frac{B_{so1}}{B}+\frac{B_{so3}}{B}}{a_1\left(a_0+1+\frac{B_{so1}}{B}+\frac{B_{so3}}{B}\right)-2\frac{B_{so1}}{B}}+\Psi\left(0.5+\frac{B_\phi}{B}\right)-\ln\left(\frac{B_\phi}{B}\right)+\right.$$

$$\left.2\ln\left(\frac{B_\phi}{B}+\frac{B_{so1}+B_{so3}}{B}\right)+\ln\left(\frac{B_\phi}{B}+\frac{2B_{so1}+2B_{so3}}{B}\right)-\sum_{n=1}^{\infty}\left(\frac{3}{n}-\frac{3a_n^2+2a_n\left(\frac{B_{so1}}{B}+\frac{B_{so3}}{B}\right)-1-2(2n+1)\frac{B_{so1}}{B}}{\left(a_n+\frac{B_{so1}}{B}+\frac{B_{so3}}{B}\right)a_{n-1}a_{n+1}-2\frac{B_{so1}}{B}[(2n+1)a_n-1]}\right)\right],$$

(5)

It is trivial to show that when B_1=0, ignoring constant shift, we recover the equation

$$\Delta\sigma(B)-\Delta\sigma(0)=-\frac{e^2}{4\pi^2\hbar}\left[-\Psi\left(0.5+\frac{B_\phi+2B_{so3}}{B}\right)-2\Psi\left(0.5+\frac{B_\phi+B_{so3}}{B}\right)+\Psi\left(0.5+\frac{B_\phi}{B}\right)-\ln\left(\frac{B_\phi}{B}\right)+\right.$$

$$\left.2\ln\left(\frac{B_\phi}{B}+\frac{B_{so3}}{B}\right)+\ln\left(\frac{B_\phi}{B}+\frac{2B_{so3}}{B}\right)\right], \qquad (6)$$

In usual analysis of these systems, linear component of SOI is ignored. Here, we found that linear component of SOI contributes to total conductance as a correction for the heterostructures with thinner STO layers. If the data does not follow the trend with strong signal to noise ratio, it might be argued that inclusion of linear term is not necessary. For the data presented in this paper, signal is strong and clean enough to warrant best possible fitting. The comparison of best fits with and without linear Rashba of the sample with 10 uc STO is given in Supplementary Fig. 6. The smoothness of the data enables us to go beyond inclusion of only cubic term (shown in of

Supplementary Fig. 6a) and can give reliable conclusion for the existence and magnitude of the linear part. To make the comparison quantitatively, we calculated the errors from both fitting models, which are obtained as the total absolute values of the differences between the calculated and the measured conductivity. As shown in Supplementary Fig. 6c, the errors from the fitting with the linear Rashba term are smaller than that without the linear term, for the samples with STO thickness less than 20 uc, while for the samples with more than 20 uc STO, the fittings with and without the linear Rashba term result in almost similar error values.

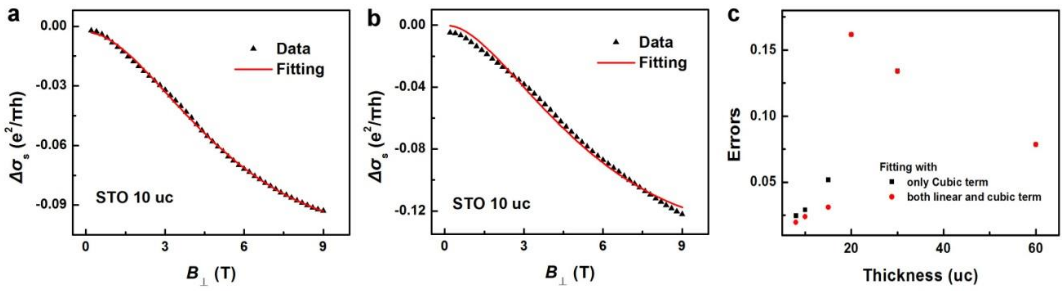

Supplementary Figure 6 | Fitting accuracy. Fitting of experimental data with (a) and without (b) linear component of Rashba in the ILP model for the LAO//STO/LAO heterostructure with 10 uc STO. Note that the experimental data appear to be different for the two cases, which is due to Lorentz force term contributing differently in the two cases. (c) shows the errors due to the fitting procedures for the heterostructures with various STO thickness.

Temperature dependent MR have been measured and fitted using the same procedures described in the main text. Supplementary Fig.7a and b shows the fitting parameters B_{so1}, B_{so3} and B_{φ} for the structures with 8 uc and 30 uc STO, respectively. For both structures, the B_{φ} increases as the temperature increases, which is consistent with the behaviour of the phase coherence property of the conducting carriers. The SOI strength, characterized by B_{so1} and B_{so3}, of both samples are not very sensitive to the temperature, except the ones at 5K of the sample with 8 uc STO. The deviation might be due to the influence of the thermal fluctuation to the quantum

correction to the conductance (The coexistence of the cubic and linear Rashba terms might be more sensitive to the thermal fluctuation).

In order to support the quantum correction scenario further, temperature dependent transport behaviour of the structures have been studied. Supplementary Fig. 8 plots the temperature dependent resistance of the samples, where significant upturns at low temperature are observed for all the structures. The inset of the figure shows the STO thickness dependent resistivity at 300K, where the resistivity is higher with thicker STO layer. The feature is consistent with the modulation effect of the carrier concentration by the STO thickness. However, it should be noted that the upturn of the resistance at low temperature range might be suppressed with the presence of the spin orbit interaction theoretically, which is not the case here. A similar phenomenon is observed in topological insulator, which is ascribed to the factors that dominate the temperature dependent behaviour, but not response significantly to the external magnetic field[9,10].

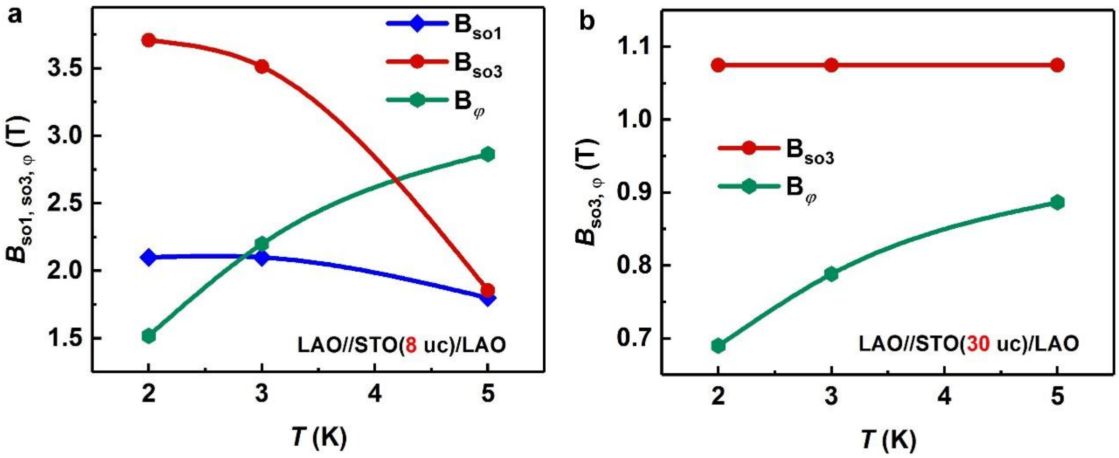

Supplementary Figure 7 | Temperature dependent fitted effective fields. (a) and (b) are for the LAO//STO/LAO structure with 8 uc and 30 uc STO, respectively.

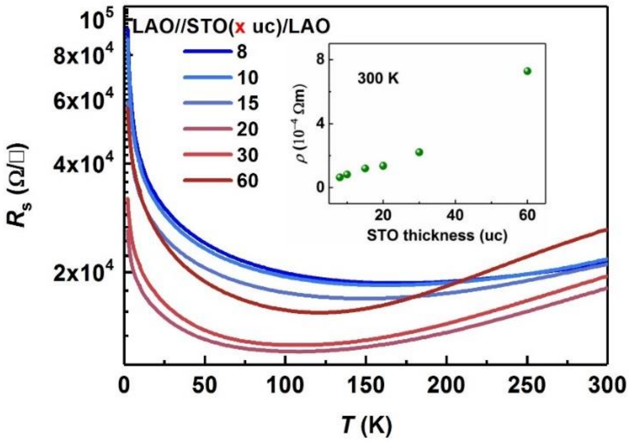

Supplementary Figure 8 | Temperature dependent sheet resistance. The R_s vs T of the LAO//STO/LAO heterostructures as functions of the thickness of the STO layer are plotted. Inset shows the thickness dependent resistivity of the structures at 300K.

Supplementary Note 5: X-ray linear dichroism (XLD) measurements of LAO//STO/LAO at Ti $L_{2,3}$-edge

Linearly polarized X-ray absorption spectroscopy (XAS) experiments were carried out by adjusting the incident angle of the X-ray beam. As shown in Supplementary Fig. 9a, the in-plane (IP) component is obtained by the normal incident X-ray, while the out-of-plane (OP) one by the grazing incident X-ray. All spectra were acquired by recording the total electron yield (TEY) at Ti $L_{2,3}$-edge. These linearly polarized X-rays will excite the electrons from the Ti 2p core level of 2p1/2 and 2p3/2 states to the unoccupied *d* orbital, and thus the intensity of the XAS (I_{IP} and I_{OP}) reflects population of the empty states. Moreover, the absorption of the X-ray shows strong dependence on the photon polarization with respect to the lattice direction, i.e. IP and OP components will excite more electrons to the d_{xy} (dx^2-y^2) and $d_{xz/yz}$ (dz^2), respectively, from the Ti 2p core level. Therefore, the sign of XLD data， (I_{IP} - I_{OP})， indicates the state of the electron occupation of the *d* orbitals in the LAO//STO/LAO structures. As shown in Supplementary Fig.

9b and c, the XLD sign at the four main peaks are positive, indicating more IP orbitals (i.e. d_{xy}) are available[11]. This means that the $d_{xz/yz}$ orbital is occupied first, which is consistent with the calculation. Such a feature with different electrons populations in different orbitals is called orbital polarization, whose strength can be characterized as $2(I_{IP} - I_{OP}) /(I_{IP} + I_{OP})$. The strengths for the structures with 30 uc STO and 8 uc STO at the t_{2g} main peak of L_3 edge are 3.50% and 4.94%, respectively, meaning more electrons occupy the $d_{xz/yz}$ orbitals at the structure with 8 uc STO. This is consistent with our Hall measurement data that the Fermi level increases as the STO thickness decreases.

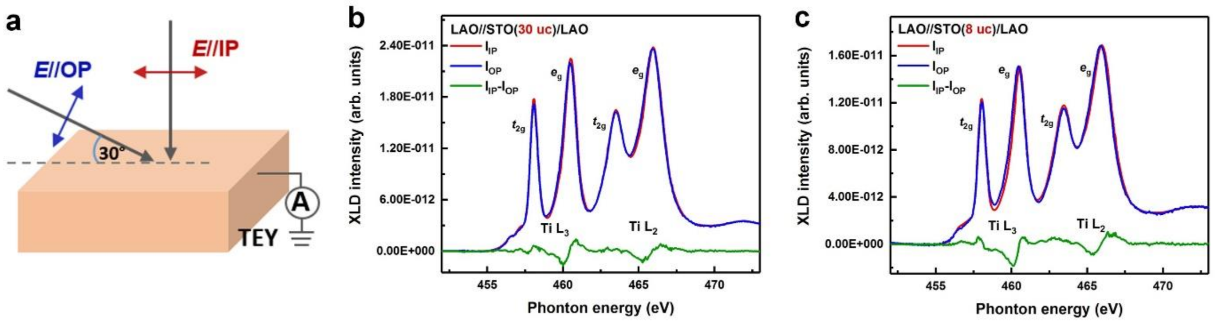

Supplementary Figure 9 | X-ray linear dichroism (XLD) measurements of LAO//STO/LAO at Ti $L_{2,3}$-edge. (a) Schematic experimental set-up for the linearly polarized X-ray absorption spectroscopy (XAS) at Ti $L_{2,3}$-edge with the total electron yield (TEY) detection mode at room temperature. In this measurement configuration, the polarization direction of the linearly polarized X-rays is achieved by modulating the X-ray incidence angle. Here, the X-ray with normal and grazing (30°) incidence correspond to the in-plane (E // IP) and majority out-of-plane (E // OP) polarized components, respectively. (b) and (c) show the XAS data for the LAO//STO/LAO structures with 30 uc and 8 uc STO, respectively. The XLD signal (green curves) are obtained from (I_{IP}-I_{OP}).

Supplementary Note 6: Calculation of the spin splitting energy

For the calculation of cubic spin-splitting energy:

$$\Delta = 2\alpha k_F^3, \quad (7)$$

where Rashba coefficient $\alpha = \frac{\hbar^2}{4\pi m^* l_{so3} n_s}$ where m^* is effective mass of electron, l_{so} is spin relaxation length, $l_{so3} = \sqrt{\hbar/4eB_{so3}}$, n_s is sheet carrier density.

And $k_F = \sqrt{2\pi n_s}$

According to the equation, for the structure with thinner STO layer involving linear Rashba, it is hard to calculate the spin splitting energy for each term, as the carrier density for each term (linear and cubic Rashba) is unknown.

Supplementary Table 1 | The derived parameters of cubic Rashba term from the magnetoconductance fitting

LAO/STO (x uc)/LAO	20	30	60
B_{so3}(T)	1.28	1.11	1.03
l_{so3} (nm)	11.3	12.1	12.6
Δ (meV)	2.87	2.60	2.30

Here $m^* = 6.8m_0$, where m_0 is the mass of electron, for orbital with cubic term.

For linear Rashba term:

$$\Delta = 2\alpha k_F, \quad (8)$$

where $\alpha = \frac{\hbar^2}{2m^* l_{so1}}$ and $k_F = \sqrt{2\pi n_s}$

From the equation above, the linear Rashba coefficient is independent of carrier density. Thus we still can get the linear Rashba coefficient for structures with thinner STO layer, but not the splitting energy, as the carrier density for the term cannot be determined.

Supplementary Table 2 | The derived parameters of linear Rashba term from the magnetoconductance fitting

LAO/STO (x uc)/LAO	8	10	15
Bso1 (T)	2.13	1.00	0.26
l_{so1} (nm)	8.8	12.8	25.0
α (eVA)	0.043	0.030	0.015

Here $m^* = 1m_0$, *where* m_0 *is the mass of electron, for orbital with linear term*

Supplementary References

1 Kim, D.-W. et al. Roles of the first atomic layers in growth of $SrTiO_3$ films on $LaAlO_3$ substrates. *Appl. Phys. Lett.* **74**, 2176 (1999).

2 Willmott, P. R. et al. Structural basis for the conducting interface between $LaAlO_3$ and $SrTiO_3$. *Phys. Rev. Lett.* **99**, 155502 (2007).

3 Cancellieri, C. et al. Electrostriction at the $LaAlO_3$/$SrTiO_3$ Interface. *Phys. Rev. Lett.* **107**, 056102 (2011).

4 Kresse, G. & Hafner, J. Ab initio molecular dynamics for open-shell transition metals. *Phys. Rev. B* **48**, 13115–13118 (1993).

5 Kresse, G. & Furthmüller, J. Efficiency of ab-initio total energy calculations for metals and semiconductors using a plane-wave basis set. *Comput. Mater.Sci.* **6**, 15–50 (1996).

6 Perdew, G et.al. Restoring the Density-Gradient Expansion for Exchange in Solids and Surfaces. *Phys. Rev. Lett.* **100**, 136406 (2008).

7 Zhong, Z et. al. Polarity-induced oxygen vacancies at $LaAlO_3/SrTiO_3$ interfaces. *Phys. Rev. B* **82**, 165127 (2010).

8 Iordanskii, S. V., Lyandageller, Y. B. & Pikus, G. E. Weak-localization in quantum-wells with spin-orbit interaction. *JETP Lett.* **60**, 206-211 (1994).

9 H. Z. Lu and S. Q. Shen, Finite-Temperature Conductivity and Magnetoconductivity of Topological Insulators. *Phys. Rev. Lett.* **112**, 146601(2014).

10 M. Liu *et al*, Electron interaction-driven insulating ground state in Bi_2Se_3 topological insulators in the two-dimensional limit. *Phys. Rev. B* **83**, 165440 (2011).

11 D. Pesquera *et al*, Two-Dimensional Electron Gases at $LaAlO_3/SrTiO_3$ Interfaces: Orbital Symmetry and Hierarchy Engineered by Crystal Orientation. *Phys. Rev. Lett.* **113**, 156802(2014).
